# Host-Associated Metagenomics: A Guide to Generating Infectious RNA Viromes

**DOI:** 10.1371/journal.pone.0139810

**Published:** 2015-10-02

**Authors:** Sarah Temmam, Sonia Monteil-Bouchard, Catherine Robert, Hervé Pascalis, Caroline Michelle, Priscilla Jardot, Rémi Charrel, Didier Raoult, Christelle Desnues

**Affiliations:** 1 Unité de Recherche sur les Maladies Infectieuses Tropicales Emergentes (URMITE) UM63, CNRS 7278, IRD 198, INSERM 1095, Aix-Marseille Université, Marseille, France; 2 Centre de Recherche et de Veille sur les maladies émergentes dans l’Océan Indien (CRVOI), IRD La Réunion, Plateforme de Recherche CYROI, La Réunion, France; 3 Emergence des Pathologies Virales (EPV), IRD 190, EHESP, Aix-Marseille Université, Marseille, France; Sidra Medical and Research Center, QATAR

## Abstract

**Background:**

Metagenomic analyses have been widely used in the last decade to describe viral communities in various environments or to identify the etiology of human, animal, and plant pathologies. Here, we present a simple and standardized protocol that allows for the purification and sequencing of RNA viromes from complex biological samples with an important reduction of host DNA and RNA contaminants, while preserving the infectivity of viral particles.

**Principal Findings:**

We evaluated different viral purification steps, random reverse transcriptions and sequence-independent amplifications of a pool of representative RNA viruses. Viruses remained infectious after the purification process. We then validated the protocol by sequencing the RNA virome of human body lice engorged *in vitro* with artificially contaminated human blood. The full genomes of the most abundant viruses absorbed by the lice during the blood meal were successfully sequenced. Interestingly, random amplifications differed in the genome coverage of segmented RNA viruses. Moreover, the majority of reads were taxonomically identified, and only 7–15% of all reads were classified as “unknown”, depending on the random amplification method.

**Conclusion:**

The protocol reported here could easily be applied to generate RNA viral metagenomes from complex biological samples of different origins. Our protocol allows further virological characterizations of the described viral communities because it preserves the infectivity of viral particles and allows for the isolation of viruses.

## Introduction

Viruses are the most ubiquitous and abundant biological entities on Earth [1]. They infect all other biological entities (such as bacteria, archaea, plants, arthropods, and mammals) living in diverse environments (such as soil, water, air, and multi-cellular organisms). Viruses influence other organisms directly by modulating their hosts’ survival via host mortality or horizontal gene transfer, or indirectly via the diversion of the host metabolic pathways during viral replication. To study viral diversity within hosts and environments, recent techniques known as viral metagenomics have emerged. Primarily based on Sequence-Independent Amplification (SIA) techniques and followed by Next-Generation Sequencing (NGS) technologies, viral metagenomics allows the description of viral communities within a complex environment without any prior knowledge of their nature. For example, in diagnostic virology, viral metagenomics have been used to identify causative viral agents of disease conditions in human [2–4] and veterinary medicine [5–7], as well as in plant [8,9] and arthropod diseases [10,11]. Virome analyses have also been conducted to describe the baseline viral diversity in healthy human conditions prior to studying the viral flora of pathologic conditions [12]. In viral ecology, metagenomics have been used to describe viral communities of diverse environments, including coastal seawater and sediment, soil, hotsprings, lakes, sewage, and air [13–17].

Viral metagenomic analyses of complex environments usually require pre-treatment steps, such as viral purification and nucleic acid enrichment, before sequencing. Several physical characteristics of viral particles enable viral purification (e.g., capsid durability), but the wide variety of viruses’ biological characteristics cause difficulties in developing a standardized protocol compatible with a broad range of particle sizes, shapes, densities, and genome types [18]. Usually, virome preparation is based on dead-end or tangential flow filtration, and nuclease digestion of non-protected viral and host cells. Then, PolyEthylene Glycol (PEG) precipitation or ultracentrifugation is eventually used, followed by nucleic acid extraction [19,20]. Although there are as many protocols to generate viral metagenomes as published metagenomic studies, the vast majority are aimed at purifying viruses from their complex matrices. This strategy, known as “Particle-Associated nucleic acid amplification”, is aimed at insolating intact (*i*.*e*., infectious) viral particles from their environment, protected from the action of nucleases [21]. Alternative steps exist within this general protocol, depending on the origin of the matrices. For example, marine biological samples such as coral tissue require chloroform homogenization of the matrix before viral purification [22].

During the last decade, several standardized protocols for generating DNA viromes from various environments have been described [18–20], but such standardization has not been reached so far for RNA viruses. Here, we present a simple protocol for the purification and sequencing of RNA viromes from host-associated biological samples of various origins. Our protocol preserves the infectivity of viral particles and allows for further applications. This protocol has been evaluated and validated by sequencing the RNA virome of body lice that were engorged *in vitro* with artificially contaminated human blood. We decided to use artificially engorged body lice as a model for host-associated metagenomics because of the ease of sampling and handling compared to human or animal specimens which require special permissions. Additionally, this system is convenient because arthropods are complex organisms in which viral, bacterial, and parasitic communities coexist.

## Materials and Methods

### Viral strains

To optimize the purification steps of the protocol, a representative panel of RNA viruses was chosen based on size, density, the presence of an envelope, and genetic composition. The latter category included whether the virus had a positive or negative strand RNA genome, and whether it contained a segmented genome. Viruses chosen were: *Yellow fever virus* 17D vaccine strain (YF), *Coxsackievirus B3* strain 2679 (CoxB3), *Influenza A/H3N2* strain Marseille/04046111/2011 (H3N2), *Cupixi arenavirus* (CPX), and MS2 bacteriophage (MS2). Additional DNA bacteriophage (T4 phage) was added to the panel to verify the efficiency of the protocol for DNA viruses and to quantify the remaining contamination of DNA in the RNA fraction of the virome (Table 1).

**Table 1:** Characteristics of reference viruses.

	YF	CPX	CoxB3	H3N2	MS2	T4
**Particle size (nm)**	50	50–300	30	80–120	26	45–230 x 825
**Density (g.cm** ^**-3**^ **)**	1.19 (sucrose)	1.17–1.18 (sucrose)	1.33–1.45 (CsCl)	1.19 (sucrose)	1.46 (CsCl)	1.50 (CsCl)
**Sensitivity to CsCl [32–35]**	yes	yes	no	yes	no	no
**Envelope**	yes	yes	no	yes	no	no
**Capsid**	icosahedral	helical	icosahedral	helical	icosahedral	icosahedral with tail
**Genome organization**	linear ssRNA, positive sense, non-segmented, 10.8 kb	linear ssRNA, ambisense, segmented (N = 2), 11 kb	linear ssRNA, positive sense, non-segmented, 7.4 kb	linear ssRNA, negative sense, segmented (N = 8), 13.5 kb	linear ssRNA, positive sense, non-segmented, 4 kb	linear dsDNA, 169 kb
**Viral family**	*Flaviviridae*	*Arenaviridae*	*Picornaviridae*	*Orthomyxoviridae*	*Leviviridae*	*Myoviridae*

YF, CPX, and CoxB3 viral strains were propagated in Vero cells while H3N2 was propagated in MDCK cells. After 4 passages, viral supernatants were nuclease-treated with 0.5 U RNAse A (Roche Diagnostics, Meylan, France) and 4 μg (1 U) of DNAse I (Sigma-Aldrich, Saint-Quentin Fallavier, France) per mL of supernatant, then precipitated with 10% PEG 8000 (Sigma-Aldrich, Saint-Quentin Fallavier, France) and 300 mM NaCl (Sigma-Aldrich, Saint-Quentin Fallavier, France) overnight at +4°C. After centrifugation at 12 000 g for 30 min at +4°C, the pellet was resuspended in 2 mL of Phosphate Buffer Saline solution (PBS), aliquoted and stored at -80°C until further use. Viral loads were estimated in Plaque-Forming Units (PFUs).

MS2 and T4 bacteriophages were purchased from the Leibniz Institute DSMZ–German Collection of Microorganisms and Cell Cultures (LGC Standards S.a.r.l., Molsheim, France) and propagated according to the manufacturer’s protocol. Bacteriophage suspensions were centrifuged at 1 500 g for 10 min, and then filtrated through 0.45 μm filters (Merck Millipore, Molsheim, France). Viral suspensions were digested with 30 U Turbo DNAse (Life technologies, Saint Aubin, France) and 25 U RNase A (Roche Diagnostics, Meylan, France) in Turbo DNase buffer at 37°C for 1 hour, then precipitated as described above. Viral loads were estimated in PFUs.

### Real-time PCR

To evaluate the efficiency of treatments for virome preparation, real-time PCRs were conducted on YF, CPX, CoxB3, H3N2, MS2, and T4 targets using the SuperScript® III Platinum One-Step RT-PCR (Life Technologies, Saint Aubin, France). The QuantiTect SYBR^®^ Green PCR/RT-PCR Kit (Qiagen, Courtaboeuf, France) was used for host-contaminating 18S DNA and RNA. Each of these reagents was used according to the manufacturers’ protocols. All quantitative real-time PCR (qPCR) and reverse transcription real-time PCR (qRT-PCR) reactions were performed in a CFX96 thermocycler (Biorad, Marnes-la-Coquette, France). YF, CoxB3, H3N2, MS2, T4 and 18S primers and probes are published in [23–27] and presented in S1 Table. CPX primers and probes were designed for this study (S1 Table). Additional primers were designed based on H3N2 and YF sequences detected in the metagenomes to verify the absence of cross-contaminations during the construction of the libraries (S1 Table). qPCR were performed using the QuantiTect SYBR^®^ Green PCR Kit (Qiagen, Courtaboeuf, France) and according to the manufacturers’ protocol.

### Ethical statement

The maintenance of a laboratory colony of *Pediculus humanus corporis* lice has been approved by the Institutional Animal Care and Use Committee of the Faculty of Medicine at Aix-Marseille University, France.

Human blood collected from healthy donors was obtained from the Etablissement Français du Sang (EFS, Marseille, France). In accordance with EFS standardized procedures for blood donation, informed consent was obtained from healthy volunteers, and personal data relative to blood donors were rendered anonymous at the time of blood donation and before blood transfer to our research lab.

### Body lice blood meal

Forty body lice were kindly provided by Jean-Michel Berenger (entomologist at “Unité de Recherche sur les Maladies Infectieuses Tropicales Emergentes”, CNRS7278, Marseille, France) and divided into two pools. Twenty lice were fed *in vitro* using artificially contaminated hemolyzed human blood, an artificial Parafilm membrane (Sigma-Aldrich, Saint-Quentin Fallavier, France) and the Hemotek system (Hemotek Ltd, Accrington, United Kingdom), an electric heating element which maintains the temperature of the blood meal, as described by Sangaré *et al*.[28]. Briefly, 5.0 x 10^10^ PFU of MS2, 1.5 x 10^9^ PFU of T4, 3.07 x 10^5^ PFU of H3N2, 1.5 x 10^4^ PFU of CoxB3, 1.0 x 10^3^ PFU of YF and 1.19 x 10^2^ PFU of CPX were added to pre-heated hemolyzed human blood to a final volume of 1.5mL, placed on the Hemotek chamber and maintained at 37°C for 30 min. During this time, lice took their blood meal. After 30 min, only 11 lice were engorged. Immediately after external decontamination of the lice, consisting of sequential washes with 10% bleach in sterile water, 70% ethanol and a final wash in sterile water, all 20 lice were stored at -80°C until further analysis. Hereafter these lice will be named “engorged lice”.

The second pool of twenty body lice was not fed with blood and was used as a positive control. Briefly, after decontamination, lice were spiked using the same concentrations of each reference virus as the ones used for blood-fed lice, and treated like the engorged ones. Hereafter these lice will be named “spiked lice”.

### Viral purification and concentration from complex samples and evaluation of the infectivity

Solid samples (*i*.*e*., “spiked” and “engorged” lice) were first homogenized in 2 mL of 0.02 μm-filtrated EMEM medium (Life Technologies, Saint Aubin, France) using the TissueLyser homogenizer (Qiagen, Courtaboeuf, France) and two 3 mm tungsten beads at 25 Hz for 2 min. Solid and liquid complex samples were then sequentially centrifuged at +4°C at 300 rpm for 30 min and 10 000 rpm for 15 min. The resulting samples were then filtered through a 0.45 μm filter (Millipore, Molsheim, France) to remove any cellular debris and bacteria.

To eliminate host DNA/RNA and free nucleic acids, 20 U Exonuclease I (New England Biolabs, Évry, France), 25 U Benzonase® (Merck Millipore, Molsheim, France), 25 U RNase A (Roche Diagnostics, Meylan, France), 20 U Turbo DNase (Life Technologies, Saint Aubin, France) and 10 μL of 10X Turbo DNase buffer were added to the clarified supernatant and incubated at 37°C for 1 hour. A total of 100 μL of the resulting supernatant was harvested to assess the concentration of Virus-Like-Particles (VLP) by fluorescence microscopy, as previously described by Thurber *et al*. [19]. All fluorescence images were acquired with a Leica SP5 inverted confocal microscope with 4 lasers, a 100 X objective and a numerical aperture of 1.4.

The resulting suspension was then deposited onto a discontinuous sucrose gradient consisting of 800 μL of a 0.02 μm-filtered layer of 66% sucrose in EMEM and 2.7 mL of a 0.02 μm-filtered layer of 30% sucrose in PBS, and ultracentrifuged in an MLS50 Beckman-Coulter rotor at 130 000 g for 2 hours at +4°C. The viral fraction was harvested from the interface between the 66% and the 30% sucrose layers using a 23G needle. The same procedure was applied for CsCl gradient ultracentrifugation to compare the maintenance of the infectivity of viral particles with sucrose gradient. Briefly, the viral suspension was deposited onto a CsCl gradient composed of 750 μL of 1.2 g/mL, 1.5 g/mL and 1.7 g/mL CsCl layers and ultracentrifuged in an MLS50 Beckman-Coulter rotor at 130 000 g for 2 hours at +4°C. The viral fraction was harvested between the 1.5 and the 1.2 g/mL CsCl layers using a 23G needle.

An aliquot of 100 μL of sucrose viral fraction of engorged and spiked lice was recovered to assess the VLP concentration by fluorescence microscopy as described above, 2 x 100 μL aliquots were immediately stored at -80°C for further viral isolation, and the resulting supernatant was used to extract nucleic acids.

Vero, MDCK and *E*. *coli* cells were used to determine the viral load of YF, CPX, CoxB3, H3N2, MS2, and T4 respectively, before and after the purification process by the lysis plaques or by the TCID_50_ methods.

### Nucleic acid extraction

Three nucleic acid extraction processes were evaluated: Trizol LS® (Life Technologies, Saint Aubin, France), the QIAmp viral RNA mini kit (Qiagen, Courtaboeuf, France), and the High Pure viral nucleic acid kit (Roche Diagnostics, Meylan, France) according to the manufacturer’s protocols. Nucleic acids were eluted in 20 μL for the QIAmp and High Pure kits and 100 μL for the Trizol LS extraction. After Trizol LS extraction, to remove any traces of phenol/chloroform that could interfere with subsequent enzymatic reactions, the RNeasy MinElute Cleanup kit (Qiagen, Courtaboeuf, France) and Agencourt AMPure beads (Beckman-Coulter, Villepinte, France) were used for the RNA and DNA fractions, respectively. Samples were then eluted in a final volume of 20 μL.

DNA fractions were used to assess the remaining host DNA contamination level. For total RNA preservation, 40 U RNase OUT (Life Technologies, Saint Aubin, France) was added.

### RNA integrity and quantification

RNA integrity was checked on an RNA6000 Pico chip (Agilent Technologies, Les Ulis, France) according to the manufacturer’s protocol, and analyzed on the Agilent 2100 Bioanalyzer.

RNA concentration was estimated with the Quanti-it Ribogreen kit (Life Technologies, Saint Aubin, France) according to the manufacturer’s recommendations, and fluorescence was quantified with the Tecan GENios fluorometer.

### RNA processing

Total RNA was processed with three different random reverse transcriptions, as previously described by Froussard *et al*. in 1992 [29], Wang *et al*. in 2002 [30], and Victoria *et al*. in 2008 [31]. Briefly, reverse transcription (RT) was conducted on 9 μL of RNA (Trizol LS extraction) or 9 μL of total nucleic acids (QIAamp and High Pure extractions) using the Superscript III Reverse transcriptase (Life Technologies, Saint Aubin, France) and the tagged-random hexamers described in the Froussard, Wang, and Victoria studies [29–31]. The thermal profile was as follows: 25°C – 5 min, 35°C – 15 min, 55°C – 30 min, and 94°C – 2 min.

Single-stranded DNA (ssDNA) was subsequently used as a template for the Klenow reaction to obtain double-stranded DNA (dsDNA). Briefly, 20 μL of ssDNA was mixed with 8 U of Klenow (Life Technologies, Saint Aubin, France) and 1 μL of 10 mM dNTP to a final volume of 30 μL. Thermal profiles were used as previously described by Froussard [29], Wang [30], and Victoria [31].

The resulting dsDNA was purified twice with the Agencourt AMPure beads (Beckman-Coulter, Villepinte, France), eluted in a final volume of 20 μL, and quantified with the Quanti-it Picogreen reagent (Life Technologies, Saint Aubin, France). Size distribution was then checked on a DNA7500 chip (Agilent Technologies, Les Ulis, France) and analyzed on the Agilent 2100 Bioanalyzer.

### RNA Sequence-Independent Amplification

For RNA sequence-independent amplification (SIA), dsDNA generated by random RT-Klenow reactions was used in random PCR. Briefly, 5 μL of dsDNA were mixed with 2.5 U of Long Amp Taq DNA polymerase (New England Biolabs, Évry, France) to a final volume of 25 μL and then randomly amplified according to Froussard [29], Wang [30], and Victoria [31]. Hereafter, the 3 viral metagenomes will be named “Froussard,” “Wang”, or “Victoria”, according to the sequence-independent amplification method used to generate it.

Amplification products were twice purified with Agencourt AMPure beads (Beckman-Coulter, Villepinte, France) according to the manufacturer’s protocol and eluted to a final volume of 15 μL. The concentration of dsDNA was estimated with the Quanti-it Picogreen kit (Life Technologies, Saint Aubin, France). Amplified products were analyzed on a DNA7500 chip (Agilent Technologies, Les Ulis, France).

### Illumina MiSeq sample preparation and processing

RNA metagenomes of engorged and spiked lice randomly amplified by “Froussard”, “Wang”, or “Victoria” random PCR were sequenced with the MiSeq Technology using paired-end and barcode strategies according to the Nextera XT library kit in a 2 x 300 bp format (Illumina Inc., San Diego CA 92121, USA). Briefly, cDNA was quantified by Qubit® with the High Sensitivity kit (Life Technologies, Carlsbad, CA, USA) and dilutions were performed to a final quantity of 1 ng of cDNA as the input. The “tagmentation” step fragmented the cDNA, and then limited cycle PCR amplification completed the tag adapters and introduced dual-index barcodes. After purification with AMPure beads (Life Technologies, Carlsbad, CA, USA), the libraries were normalized on specific beads according to the Nextera XT protocol (Illumina Inc., San Diego CA 92121, USA). Normalized libraries were sequenced along with 11 other projects for a total of 18 projects. Automated cluster generation and paired-end sequencing with dual-index reads were performed in a single run of 2 x 300 bp read length.

### Sequence processing and virus genome identification

Paired reads were imported into the CLC Genomics Workbench 6.0.1 program (CLC Bio, Aarhus, Denmark) with importing parameters including minimum and maximum distances set at 50 and 400, respectively. Raw Illumina reads were first trimmed according to their quality score (Illumina pipeline 1.8 and later), their length (reads < 50 nt long were discarded) and according to the primers used for random PCR.

Reads were then mapped onto reference genomes using the CLC Genomics Workbench 6.0.1 program (CLC Bio, Aarhus, Denmark) with mapping parameters that included a minimal length fraction of 0.5, a minimal similarity fraction of 0.8, a mismatch cost of 2, and an insertion/deletion cost of 3. The GenBank accession numbers of YF, CPX, CoxB3, H3N2, MS2, and T4 reference genomes used for mapping are JN628279, AY216519, AY896763, CY114421, V00642, and AF158101, respectively.

Un-mapped reads were *de novo* assembled into contigs using the CLC Genomics Workbench 6.0.1 assembler with stringent assembly parameters: minimal length fraction of 0.9, minimal similarity fraction of 0.75, word size of 2, minimal contig length of 200 bp, mismatch cost of 2 and insertion/deletion cost of 3. Contigs and singletons of un-mapped reads were compared to the NCBI nucleotide database using the BlastN program, with a minimum coverage of 50%, a minimum identity of 50%, and an E-value < 10^−5^. Reads and contigs having no significant hits according to the criteria were classified as “unknown”. Table 2 presents the data from the Illumina MiSeq sequencing of engorged and spiked lice according to the random PCR method.

**Table 2:** Data of the Illumina MiSeq sequencing of engorged and spiked lice according to the random PCR method.

ENGORGED LICE	“Froussard”	“Wang”	“Victoria”
**Total number of reads (R1+R2)**	**1,594,552**	**1,673,532**	**1,181,724**
Total mapped reads	721,520	697,950	716,305
Un-mapped reads	873,032	975,582	465,419
Contigs of un-mapped reads	1,262	473	335
Singletons of un-mapped reads	270,157	226,773	130,193
**Total assigned reads**	**1,360,372**	**763,442**	**1,097,434**
**Viral reads:**	**728,700**	**749,544**	**738,929**
MS2 reads	617,744	595,402	678,268
CoxB3 reads	107,252	150,502	60,619
H3N2 reads	181	3,552	18
T4 reads	8	0	2
YF reads	0	0	0
CPX reads	0	0	0
**Other viral reads:**	**3,515**	**88**	**22**
*Podoviridae* (T7 and T3 phages)	3,477	56	6
*Leviviridae* (BO1 phage)	1	0	0
*Siphoviridae* (*Staphylococcus* phage phiETA2)	0	1	0
*Retroviridae* (Squirrel monkey retrovirus-H)	37	31	11
*Mimiviridae* (Mimivirus and Mamavirus)	0	0	5
**Eukaryote reads**	**618,992**	**657,100**	**355,448**
**Prokaryote reads**	**9,165**	**7,239**	**3,035**
**Unknown reads**	**234,180**	**910,090**	**84,290**
**SPIKED LICE**	**“Froussard”**	**“Wang”**	**“Victoria”**
**Total number of reads (R1+R2)**	**1,460,831**	**1,917,827**	**1,673,347**
Total mapped reads	1,442,668	1,639,590	1,636,226
Un-mapped reads	18,163	278,237	37,121
Contigs of un-mapped reads	381	275	145
Singletons of un-mapped reads	6,208	26,988	873
**Total assigned reads**	**1,457,577**	**1,629,308**	**1,654,195**
**Viral reads:**	**1,447,878**	**1,609,176**	**1,644,755**
MS2 reads	1,433,365	1,528,259	1,622,373
CoxB3 reads	6,848	77,161	21,488
H3N2 reads	2,439	3,486	749
YF reads	14	64	4
CPX reads	2	2	2
**Other viral reads:**	**1,627**	**204**	**139**
*Podoviridae* (T7 and T3 phages)	899	734	65
*Microviridae* (phiX174 phage)	12	4	7
*Retroviridae* (Squirrel monkey retrovirus-H)	713	4,432	67
*Mimiviridae* (Hirudovirus Sangsue, Samba virus)	2	3	0
**Eukaryote reads**	**4,742**	**16,543**	**7,849**
**Prokaryote reads**	**4,957**	**3,589**	**1,591**
**Unknown reads**	**3,254**	**288,519**	**19,152**

## Results

### Viral particles purification and concentration

**Fig 1:**
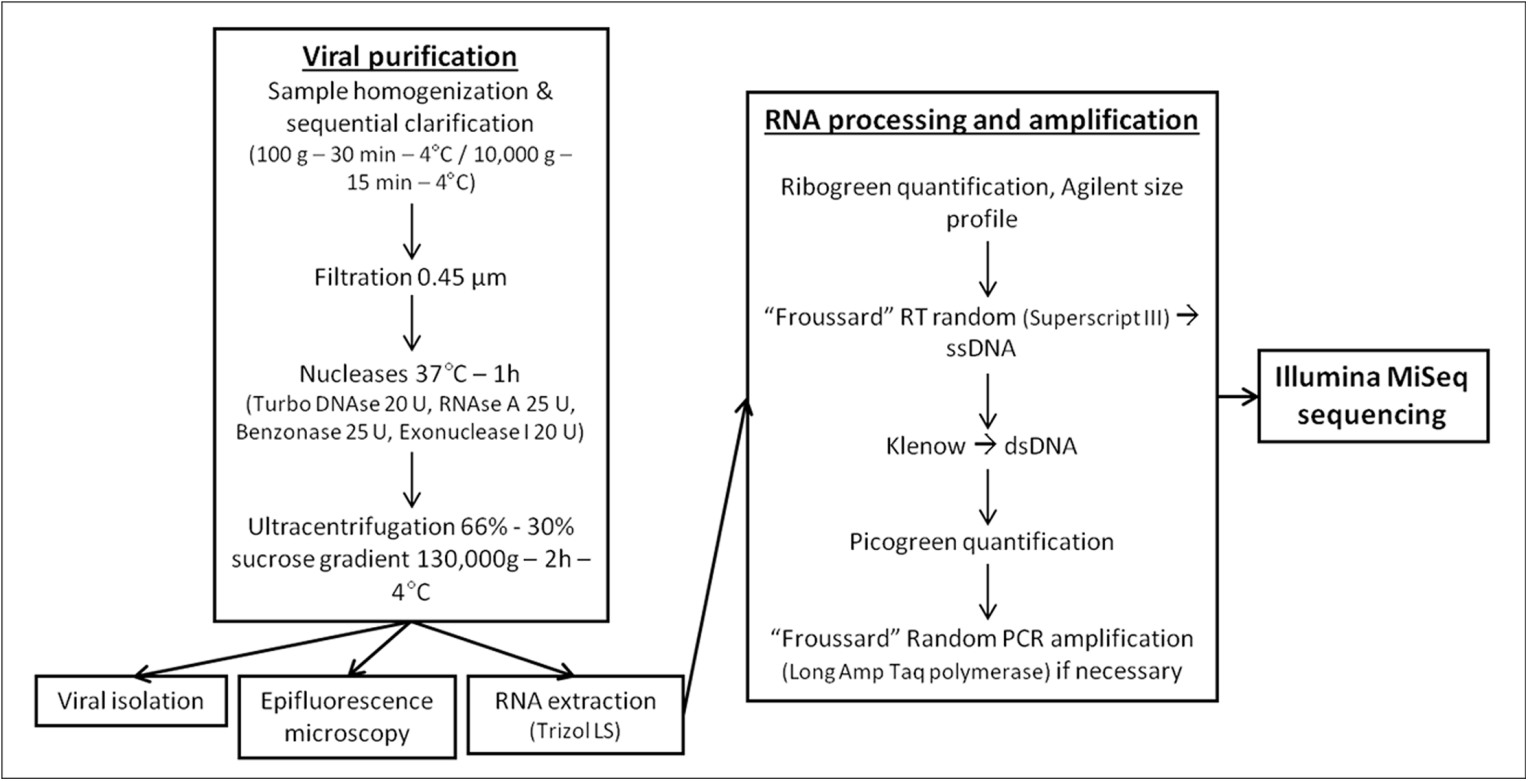
General overview of the protocol.

When studying viral communities of complex environmental or biological samples, diverse eukaryote, prokaryote, and archaea communities may interfere with the analysis. As a consequence, several pretreatments are required to purify the virome from these contaminants, mainly based on the difference in size and density of viral particles compared to those of eukaryotic and prokaryotic cells. A general overview of the process developed here is presented in Fig 1. To assess the loss of contaminants during the whole purification process, qPCR and qRT-PCR targeting the 18S DNA and RNA, respectively, were performed before and after each step of the process. Similarly, control real-time RT-PCR and PCR targeting the reference RNA and DNA viruses, respectively, were conducted to verify their presence after each step of the purification process.

The first step was a 0.45 μm filtration followed by nuclease digestion. The filtration eliminated 0.6 log_10_ and 0.4 log_10_ of 18S DNA and RNA, respectively, and the nuclease treatment eliminated an additional 1.8 log_10_ and 2.1 log_10_ of 18S DNA and RNA, respectively. Interestingly, the use of the nuclease cocktail, *i*.*e*., Turbo DNAse–RNAse–Benzonase–Exonuclease I, resulted in better elimination of 18S DNA and RNA contamination than the use of Turbo DNAse and RNAse in combination. Indeed, the Ct value of the 18S DNA contamination was 36.38 with the use of Turbo DNAse–RNAse but negative with the use of the nuclease cocktail. Moreover, the 18S RNA contamination was eliminated more effectively with the nuclease cocktail than with the use of the Turbo DNAse–RNAse combination (Ct = 34.85 vs 32.67, respectively). No difference was observed in the 18S contamination when samples were incubated with the nuclease cocktail for between one and two hours (ΔCt between one and two hours of nuclease digestion of 0.48 for 18S DNA and 0.29 for 18S RNA). The reference DNA and RNA viruses were not affected by filtration and nuclease treatments (with ΔCt values ranging from 0.15 to 1.33 depending on the virus).

To increase the elimination of 18S DNA and RNA contamination via ultracentrifugation, two discontinuous sucrose gradient formulations were tested (66%-20% and 66%-30%). For a given centrifugation speed and time, the 66%-30% sucrose gradient was more effective at removing the 18S contamination, with less than one log_10_ of loss of viral load for the reference DNA and RNA viruses. There was no major difference observed between the two sucrose gradients for the removal of 18S DNA contamination (-3.37 log_10_ for the 66%-30% gradient and -2.98 log_10_ for the 66%-20% gradient); however, the difference was important for the 18S RNA contamination, as 0.20 log_10_ of 18S RNA was removed with the 66%-20% sucrose gradient versus more than 3 log_10_ of 18S RNA using the 66%-30% sucrose gradient. Additionally, the reference viruses were not affected by the increase in sucrose density (-0.46 log_10_ for YF, -0.00 log_10_ for CPX, -0.18 log_10_ for CoxB3, -0.67 log_10_ for H3N2, and -0.00 log_10_ for T4 when increasing the sucrose density from 66%-20% to 66%-30%).

**Fig 2:**
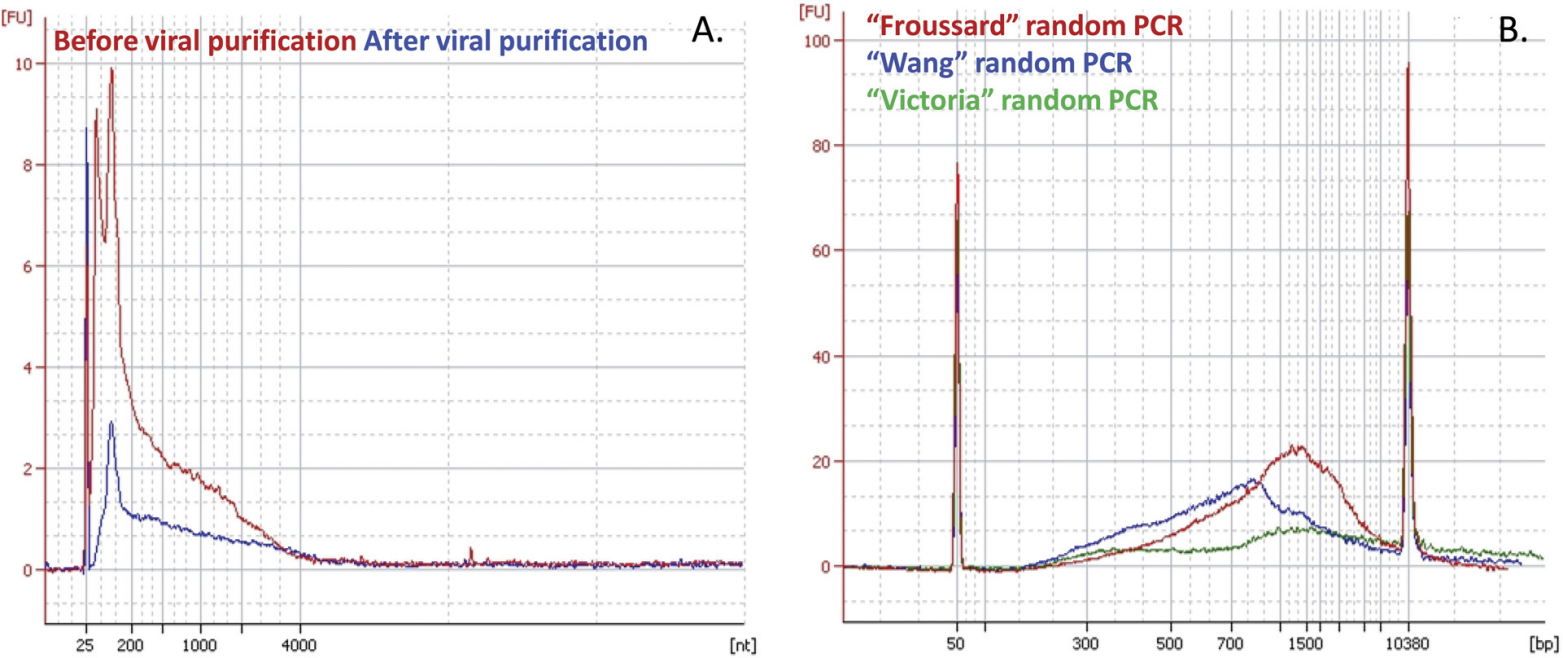
Nucleic acid profiles analyzed on a 2100 Expert Agilent Analyzer. A. RNA profile of an EMEM sample spiked with the reference viruses before (red) and after (blue) viral purification, run on a Pico RNA chip. B. DNA profile of a dsDNA sample (originated from RNA) amplified either with Froussard (red), Wang (blue) or Victoria (green) random PCR, run on a DNA7500 chip.

Fig 2A presents the RNA profile of an EMEM sample spiked with the reference DNA and RNA viruses pretreated by a 0.45 μm filtration followed by nuclease digestion and ultracentrifugation, before and after viral purification. Although a clear decrease in the total amount of RNA was observed, which reflected the loss of 18S RNA, small RNAs (between 100 and 200 nt) were detected. This could suggest the recovery of digested host RNAs, despite the nuclease treatment and ultracentrifugation step (Fig 2A).

Further optimizations, primarily of the ultracentrifugation step, were tested to improve the purification process but did not achieve satisfying results. Briefly, (i) an increase in centrifugation speed from 130 000 g to 160 000 g did not improve the recovery of reference viruses (ΔCt ranging from 0.26 to 0.91, depending on the reference virus), but did result in the recuperation of more contaminating 18S RNA (loss of total 18S RNA estimated at 6.78 Ct when ultracentrifuged at 130 000 g and only at 0.56 Ct when ultracentrifuged at 160 000 g); (ii) the addition of a final pelleting ultracentrifugation step after the discontinuous gradient ultracentrifugation step resulted in an important loss of infectivity of reference viruses and their increased sensitivity to nucleases; (iii) the addition of a final nuclease digestion step after ultracentrifugation, to remove any remaining 18S DNA and RNA contaminant, resulted in a major loss of 18S DNA and RNA contamination (negative PCR for 18S DNA and RNA after a final nuclease treatment but a resulting Ct = 32.91 and Ct = 30.44, respectively, if no final nuclease step was added). However, we observed a concomitant loss of several viral reference genomes (negative PCR for CoxB3 and H3N2 if a final nuclease digestion is performed whereas Ct values of 22.20 and 29.42, respectively, with no final nuclease treatment).

### Maintaining viral infectivity

For the ultracentrifugation step, the choice of sucrose gradient instead of cesium chloride (CsCl), usually used to generate viral metagenomes, was motivated by the wish to preserve the infectivity of viral particles. Indeed, the use of CsCl gradients alters the integrity of enveloped virions and affects their infectivity (Table 1) [32–35]. Consequently, we tested the infectivity of recovered viral particles after all purification processes by re-isolating viruses after the purification steps, either in 66%-30% sucrose or in 1.7–1.5–1.2 g/mL CsCl gradients. The resulting cytopathic effects are presented in S1 Fig. After two days of culture, sucrose and CsCl revealed a toxic effect on Vero cells, with higher cytotoxicity for CsCl than for sucrose. Indeed, 1/10 and 1/100 dilutions were necessary to inhibit cell toxicity for sucrose and CsCl, respectively (S1A Fig).

For non-enveloped viruses (*i*.*e*. MS2 and CoxB3), no difference was observed between the viral titer obtained after either sucrose or CsCl purification, suggesting that the infectivity of virions was preserved. Indeed, for MS2 bacteriophage, we estimated the resulting loss of viral titer after the process to be -0.86 log_10_ after the sucrose gradient step and -0.80 after the CsCl gradient step; for CoxB3, the resulting loss of viral titer was estimated at -1.12 log_10_ after the sucrose gradient step and -1.00 after the CsCl gradient step.

Conversely, cytopathic effects due to CPX and YF infections were observed only after sucrose purification (S1B Fig), confirming the alteration of viral structures and the loss of infectivity after CsCl ultracentrifugation and the recovery of infectious viral particles after 66%-30% sucrose gradient.

### Nucleic acid extraction

Three nucleic acid extractions processed on an EMEM sample spiked with the reference viruses were evaluated according to the extraction yield and the degradation of RNA: Trizol LS, QIAmp viral RNA mini kit from Qiagen, and High Pure viral nucleic acid kit from Roche. Although no difference was observed in the extraction yield between Qiagen and Trizol LS (13.5 ng/μL and 19.8 ng/μL of total RNA, respectively), Roche extraction did not reach the same yield (3.1 ng/μL of total RNA). The RNA profile of the 3 extracts presented the same small RNAs as presented in Fig 2A (*i*.*e*., those between 100 nt and 200 nt), suggesting a degradation of nucleic acids during the extraction process.

Although real-time PCR targeting the DNA and RNA reference viruses conducted on these 3 extracts revealed an average difference of one log_10_ (3 Ct) between Qiagen (the best), Trizol LS, and Roche, Trizol LS extraction was chosen because of its capacity to extract large sample volumes (> 1 mL), as recovered after the sucrose gradient ultracentrifugation step. Moreover, the Trizol LS extraction allowed for separate extraction of DNA and RNA, while Qiagen and Roche both extracted total nucleic acids and therefore required a DNAse post-extraction treatment for the RNA virome preparation.

### RNA processing and random PCR

Hereafter, the 3 metagenomes are designated as “Froussard”, “Wang”, or “Victoria”, according to the sequence-independent reverse transcription and amplification methods used to generate them.

Total RNA was processed in three different random reverse transcriptions (RT), as previously described by Froussard [29], Wang [30], and Victoria [31]. For each random RT, different random primer concentrations were tested to determine the best concentration for generating cDNA fragments with lengths compatible with Illumina MiSeq requirements. For Froussard RT, we tested 0.001 μg/μL, 0.01 μg/μL, 0.05 μg/μL, 0.10 μg/μL (published concentration), and 0.15 μg/μL of primer. For Wang RT, we tested 0.4 pmol, 4 pmol, 20 pmol, 40 pmol (published quantity), and 60 pmol of primer. For Victoria RT, we tested 1 pmol, 10 pmol, 50 pmol, 100 pmol (published quantity), and 150 pmol of primer. No difference was observed regarding the size of cDNA fragments when increasing or decreasing the quantity of random primer compared with the published primer concentration used for reverse transcription. The effect of different random primer concentrations used in the reverse transcription step on the size of the generated amplicons is presented in S2A–S2F Fig.

For RNA sequence-independent amplification, dsDNA generated by random RT-Klenow reactions was used in random PCR. To minimize the amplification step and the potential resulting bias of sequence representation in the metagenome, three cycling conditions were tested for each random PCR: 10, 20, and 40 cycles. No difference in dsDNA size profile was observed between 20 and 40 PCR cycles, but difference was observed in the yield of amplification. Ten cycles of amplification failed to reach the minimum amount of dsDNA material required by Illumina MiSeq whereas 20 and 40 cycles of amplification produced a sufficient quantity. For example, a cDNA sample quantified at 0.23 ng/μL was amplified at the yield of 0.27 ng/μL, 0.42 ng/μL, and 84.06 ng/μL after 10, 20 and 40 cycles of Froussard random amplification, respectively. Although only 20 cycles were enough to obtain a sufficient quantity of dsDNA for several samples (at least 1 ng, according to Illumina recommendations), we decided to use 40 cycles in all cases to ensure enough sequencing material and to ensure similar sample treatments. Fig 2B presents the DNA profile of a dsDNA sample amplified with Froussard, Wang, or Victoria PCR after 40 cycles of random amplification. A difference in size profiles of dsDNA between the amplification methods should be noted: 500–2 000 bp with a maximum at 800 bp for Wang amplification, and 300–10 000 bp with a maximum at 1 500 bp for Froussard and Victoria random PCRs. As a result, users can adapt random amplifications to their sequencing technology requirements. For example, a shorter elongation step in the Froussard random amplification results in the generation of smaller fragments, which are compatible with Illumina MiSeq or Roche 454 requirements (S2H Fig).

Finally, we compared the effect of the use of different random PCR on the amplicon size profile for a given reference virus. For each tested reference virus, Froussard, Wang or Victoria amplification methods generated different amplicon sizes (S2I–S2L Fig), revealing the amplification bias when SIA is performed.

### Protocol validation: artificial arthropod RNA virome

To evaluate the protocol on a complex sample, twenty body lice were fed *in vitro* with human blood supplemented with YF, CPX, CoxB3, H3N2, MS2, and T4 viruses, and then further processed with the methodology for RNA virome preparation and analysis described above. T4 DNA bacteriophage was added to the RNA viruses’ panel in order to assess the residual DNA contamination of the RNA fraction. Twenty non-engorged body lice were concomitantly used as a positive control, *i*.*e*. they were spiked with the same amount of viruses as that used for engorged lice, and further processed and sequenced as previously described. Viral concentrations applied to either fed- or spiked-lice followed a Gaussian distribution that mimics “natural” conditions. This range of concentrations was also used to evaluate the sensitivity of the virome protocol.

We first evaluated by qRT-PCR and qPCR the loss of viral particles during the purification process of the positive control. A difference of less than one log_10_ was observed for YF (ΔCt = 2.1) and CoxB3 (ΔCt = 2.6), more than one log_10_ for H3N2 (ΔCt = 4.06), and no difference for MS2 before and after the purification process, suggesting that the process did not affect the viral load of the reference viruses. For T4 DNA phage, a difference of less than one log_10_ (ΔCt = 1.2) was observed before and after the purification steps. CPX was negative, both before and after the purification process, probably due to a low viral load and a high limit of detection of the qRT-PCR system. We then purified this artificial positive control in the same way as the engorged lice, as described hereafter.

After homogenization of the engorged lice, clarification and filtration through a 0.45 μm filter, the clarified homogenate was treated with a cocktail of DNA and RNA nucleases to remove the majority of host DNA/RNA contaminants. To purify viral particles from their complex environment, the supernatant was ultracentrifuged on a 66%-30% sucrose gradient, and total RNA were extracted from the resulting interface using the Trizol LS® extraction method. To control the recovery of viral particles and the efficiency of viral purification, fluorescence microscopy was conducted on an aliquot of the purified viral fraction (S3 Fig). No particles with a size compatible to that of bacterial and eukaryotic cells were observed, suggesting good recovery of viral particles after treatments.

Quantification estimated 1.3 ng/μL of total RNA in the engorged lice extract. To evaluate the remaining host DNA and RNA contamination, real-time 18S PCR and RT-PCR were conducted on RNA extracts, resulting in a Ct = 28.85 for the DNA 18S PCR and a negative result for the RNA 18S RT-PCR. These results highlight the presence of a residual host DNA contamination in the RNA fraction. Positive qRT-PCR signals were detected for MS2 (Ct = 15.10) and CoxB3 (Ct = 23.02) viruses, but not for YF, CPX, and H3N2 viruses amplified with published primers.

RNA extracted from engorged and spiked lice was further processed with the Froussard, Wang, or Victoria random reverse transcription-Klenow reactions described above. The resulting dsDNA was quantified using the Quanti-it Picogreen, but quantifications were negative. The low amount of nucleic acid material required the use of random PCR. Only 40 cycles of random PCR achieved the quantity of DNA compatible with Illumina MiSeq requirements.

Table 2 presents the data of the Illumina MiSeq sequencing according to the random PCR method for both engorged and spiked lice. After MiSeq sequencing, with a run of 2 x 300 bp read length, 7.64 Gb of total information was obtained from a 524 K/mm^2^ cluster density with 12,380,000 passed filter paired reads (96.1% of clusters passing the quality control filter). Within this pooled run, the index representation for the 6 cDNA samples ranged from 4.8% to 7.8%, corresponding to 590,972 to 969,911 passed filter paired reads.

**Fig 3:**
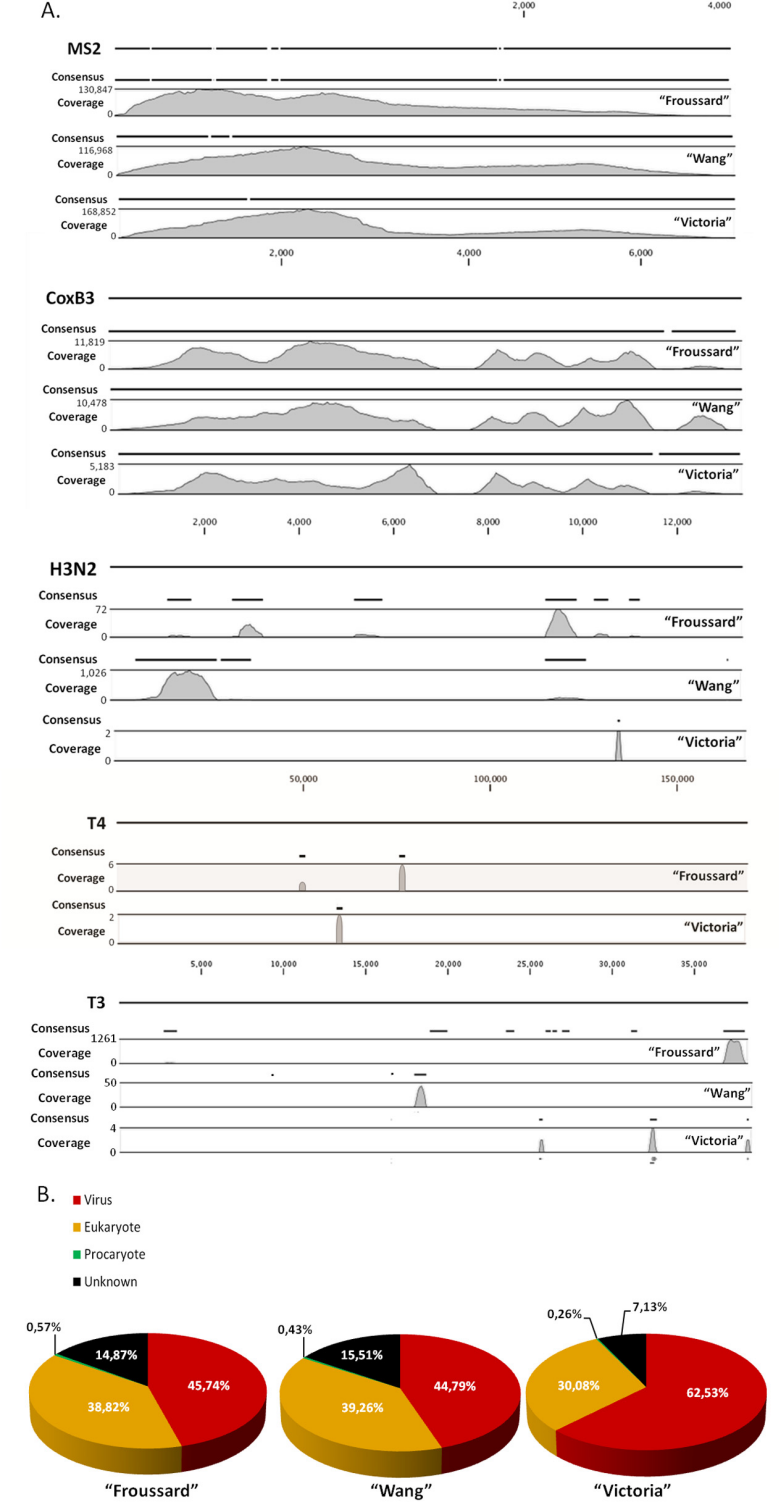
Comparison of the 3 random PCR reactions in engorged lice metagenomes according to: A. reference genome coverage. B. General taxonomic assignment of reads.

Bioinformatics analyses of the Froussard, Wang and Victoria engorged lice metagenomes are presented in Fig 3 and in Table 2. The mapping of total reads against reference genomes of YF, CPX, CoxB3, H3N2, MS2, and T4 are presented in Fig 3A. Results of mapping and taxonomic assignation of reads of the positive control (*i*.*e*. spiked lice) RNA metagenome are presented in Table 2 and S4 Fig. No difference was observed in the genome coverage for MS2 and CoxB3 between the 3 engorged lice and the 3 spiked lice RNA viromes. Coverage was 99.41% for MS2 and 97.45% for CoxB3 in the Froussard engorged metagenomes, 99.30% for MS2 and 100% for CoxB3 in the Wang engorged metagenomes, and 99.41% for MS2 and 98.56% for CoxB3 in the Victoria engorged metagenomes. T4 qPCR was negative and very few reads of T4 DNA bacteriophage were obtained in the RNA Froussard and Victoria metagenomes. However, several reads of T3 DNA bacteriophage were obtained, either for engorged or spiked lice metagenomes (Table 2, Fig 3, and S4 Fig). A recent study has shown that the T4 strain (11303-B4) provided by the ATCC was indeed characterized as a T3 strain after sequencing [36]. Since we spiked the blood with the same strain and since our results from sequencing showed only few reads related to T4 compared to the high abundance of T3 phage reads, we believe that the T3 reads recovered derived from T3 phage spiked in the blood sample. These results thus indicate a remaining viral DNA contamination of the RNA fraction after Trizol LS® extraction (even after a second Trizol/chloroform purification performed in the RNA aqueous phase). Sequencing of H3N2 in the RNA metagenome yielded coverage of 22.21%, 24.21% and 0.40% for Froussard, Wang and Victoria, respectively, after the body lice blood meal following inoculation of 3.07 x 10^5^ PFU of H3N2 in the blood meal. Although a negative qRT-PCR result was obtained for H3N2 in the lice extract using primers from the literature, amplification using primers specifically designed on H3N2 reads recovered after sequencing was positive (data not shnown), confirming the presence of H3N2 viruses in the lice. The discrepancies observed between PCR results obtained with published or virome-based primers are likely due to differences in primers sensitivities. Additionally, no reads were obtained for YF and CPX, which were also negative after qRT-PCR (both with published and virome-based primers), most likely due to the low amount of viral particles ingested by the lice during their blood meal (less than 1.0 x 10^3^ PFU of YF and 1.19 x 10^2^ PFU of CPX added to the blood). Indeed, as described by Cheval *et al*. in 2011 [37] and by Frey *et al*. in 2014 [38], the limit of detection of next-generation sequencing techniques are estimated at 10^3^ to 10^4^ genome copies per mL.

Sequencing of the spiked lice resulted in the same pattern of viral relative abundances and coverage as the one observed for engorged lice (Table 2, S4 Fig), suggesting that the difference in the observed read abundances, either for the engorged or for the spiked lice, did not result from the purification process and was probably due to intrinsic characteristics of the reference viruses. Interestingly, whereas no reads of YF and CPX were detected in the engorged lice metagenomes, whatever the amplification method, a few reads of YF and CPX were detected in the spiked lice metagenomes (N = 2 to 32 reads for YF, depending on the amplification method, N = 1 read for CPX, whatever the amplification method) (Table 2). YF was positive in qRT-PCR both with the published and virome-based primers after the process, and CPX was negative, suggesting that the viral load of these viruses was probably at the limit of detection of the sequencing method (*i*.*e*. between 10^3^ PFU [as for YF] and 10^2^ PFU [as for CPX]).

No difference was observed in the depth of sequencing. The average genome coverage of MS2 and CoxB3 was estimated at 46,035 and 3,707, respectively, for Froussard engorged lice metagenomes, 43,559 and 3,495, respectively, for Wang engorged lice metagenomes, and 50,193 and 1,344, respectively, for Victoria engorged lice metagenomes. The main difference between the 3 engorged lice metagenomes was in H3N2 mapping: the Wang random PCR seemed to amplify preferentially large segments (PB2 and PB1 polymerase segments), while the Froussard method seemed to randomly amplify all segments (Fig 3A). Interestingly, this bias of repartition of reads along the whole genome of H3N2 was not observed in the positive control (S4 Fig), suggesting that with larger amounts of viruses, Wang and Victoria SIA are able to randomly amplify each segment of the virus. Nevertheless, in “environmental” conditions (*i*.*e*. with low viral loads), only the Froussard SIA seemed to be able to randomly amplify each segment of segmented viruses (Fig 3A).

There was no notable difference in the taxonomic assignment of reads when comparing the 3 engorged lice metagenomes (Table 2). Froussard, Wang, and Victoria metagenomes were able to identify 85.13%, 84.49%, and 92.87% of reads, respectively. Of these, viral reads represented 45.74% (721,520 reads) of Froussard, 44.79% (749,587 reads) of Wang, and 62.53% (38,939 reads) of Victoria metagenomes; eukaryote reads represented 38.82%, 39.26%, and 30.08%, respectively, and prokaryote reads (bacteria and archaea) represented only 0.57%, 0.43%, and 0.26% of the respective total reads (Fig 3B). Despite all the pretreatments applied to decrease the host contamination, human and arthropod sequences persisted. Indeed, human contamination of the viromes was estimated at only 0.67%, 0.17%, and 0.044% for Froussard, Wang, and Victoria metagenomes, respectively, but remaining arthropod contamination was estimated at 38.05%, 39.05% and 29.98%.

## Discussion

Viral metagenomics has been used worldwide to describe viral communities in various complex environments [13–17] or to identify the etiology of human, animal, and plant pathologies [2–9]. This work resulted in the generation of several matrix-dependent methods to generate viromes. In this study, we aimed at developing a standardized protocol for the purification and sequencing of RNA host-associated viromes from complex biological samples of different origins that would preserve the infectivity of viral particles and allow for further virological characterizations, with an important reduction of host DNA/RNA contaminants in order to fully benefit the depth of sequencing of NGS techniques.

The RNA metagenome preparation protocol is based primarily on the “particle-associated nucleic acid amplification” strategy [21]. That is, it tries to purify intact viral particles from their environment, protected from the action of nucleases used to degrade host-contaminating DNA and RNA. The first step of the protocol is the filtration of the supernatant using 0.45 μm filters. Most previous metagenomic studies using filtration as a purification step used filters with a pore size of 0.22 μm [39,40], however the use of a 0.45 μm filtration step allows the recovery of large DNA viruses whose size would not permit recovery with smaller pore size filters [41].

To eliminate the majority of contaminating host DNAs and RNAs, various strategies are used, such as soft cell lysis, which leaves nuclei intact [42], but the remaining nucleic acids from hosts require nuclease post-treatment. Here we propose the use of a combination of Exonuclease I, Benzonase, RNase A and Turbo DNase to enhance the digestion of host DNA and RNA compared to the use of only RNAse A and Turbo DNAse. Several studies reported that the use of RNAse A in combination with proteinase K could result in the inactivation of some viruses, such as MS2 bacteriophage [43], if no further inactivation of nucleases and proteases is performed. In our protocol, to prevent the inactivation of several particles, which would result in a significant loss of infectivity, the digested supernatant was immediately loaded onto a sucrose gradient to purify viral particles from the action of nucleases.

The next purification step was ultracentrifugation on a discontinuous 66%-30% sucrose gradient. A broad range of methods has been described to purify and concentrate viral particles. These include tangential flow filtration (TFF) [40,44,45], PEG precipitation [18,19,46], cesium chloride (CsCl) gradient ultracentrifugation [22,32,44], and more recently iodixanol (Optiprep^TM^) density gradient medium. The main disadvantage of CsCl ultracentrifugation is that when analyzing the viral communities of an environment, the lack of prior knowledge of the viruses constituting these communities prevents the harvesting of specific fractions where viruses are located, resulting in recovery of all fractions and thereby diluting the viruses. Iodixanol gradient ultracentrifugation is used either in continuous or in isopycnic gradient to purify viral particles, resulting, as for CsCl, in the necessity to harvest all viral fractions and further diluting the viruses, unless the density of a specific virus is known [47]. Further pelleting by ultracentrifugation would overcome this problem, but we have shown that such a pelleting would damage the integrity of viral particles (recovered viruses were sensitive to nucleases used in a final step, suggesting that the structure of the virion was affected by the two ultracentrifugations). PEG precipitation would also overcome this problem, but would usually result in the precipitation many host DNA, RNA, and protein contaminants.

Recently, a protocol for virome preparation has been published by Kohl *et al*. [48] in which 80%-20% sucrose gradient is used to purify viral particles. The authors used a second ultracentrifugation step to pellet viruses harvested after the gradient ultracentrifugation. In our protocol, we noted that (i) the use of a discontinuous 66%-30% sucrose layer enhanced the purification of viral particles from host nucleic acid contaminants and (ii) a further pelleting ultracentrifugation step followed by nuclease treatments would damage the integrity of virions and increase their sensitivity to nucleases. Here we propose a single ultracentrifugation step based on a discontinuous 66%-30% sucrose gradient. Even if most viruses displayed large differences in their physical-chemical properties, most of them would migrate at the interface between the two sucrose layers. This would result in a single “ring” for recovery, usually < 1 mL and compatible with most nucleic acid extraction protocols.

One key step of metagenome preparation concerns total nucleic acid extraction. Indeed, the assessment of the description of viral diversity of an environment, as well as the genome coverage of viruses, depends on the quality and quantity of extracted nucleic acids. Usually, column-based extraction kits allow for preferential extraction of either DNA or RNA, or both in the same tube, which leads to the splitting in two of the extracts to generate separate DNA and RNA viromes. Recently, column-based kits allowing the extraction of separate DNAs and RNAs from a single sample tube have emerged, but a recent study conducted by Mathieson *et al*. concluded that the quality and integrity of the nucleic acids isolated were compromised using these kits [49]. For this reason, we chose the Trizol LS® system. However, using a DNA bacteriophage as a control of the remaining DNA contamination of the RNA virome, we demonstrated that RNA Trizol LS® extraction allows for the recovery of at least some viral DNA, even if two consecutive extractions of the aqueous phase are performed. Additionally, we selected this reagent for its capacity to extract DNA and RNA separately from large sample volumes (> 1 mL), such as those generated after the recovery of the viral interface resulting from the ultracentrifugation step.

Usually, extraction yields of DNA and RNA obtained after all the viral purification steps are not sufficient for direct sequencing, and they often require a sequence-independent amplification. DNA SIA is biased [50,51] but quite easy, mainly based on the use of phi29 DNA polymerase through Multiple Displacement Amplification (MDA) or Rolling Circle Amplification (RCA) [52]. However, the sequence-independent amplification of RNAs requires several pre-processes to reverse transcript RNA in cDNA before the amplification. For RNA SIA, several amplification methods have been described and compared, including Sequence-Independent Single-Primer Amplification (SISPA), Degenerate Oligonucleotide-Primed PCR (DOP-PCR), and random PCR [52,53]. Although these techniques allow the generation of enough nucleic acid material for sequencing, their main disadvantage remains that they distort quantitative analyses by introducing bias of amplification in viral diversity studies. A recent study published by Karlsson *et al*. concluded that SIA introduces a strong amplification bias, consisting of inhomogeneous genome coverage and sequence depth [54]. These problems are probably due to the tag sequence, as reported by Rosseel and collaborators [55]. In fact, in the present study similar results were obtained when comparing the genome coverage of reference viruses after Froussard [29], Wang [30], and Victoria [31] random amplifications. These 3 techniques only differ in the tag sequences and the lengths of the 3’ random hexamers. Although no notable differences were observed for MS2 and CoxB3 genome coverage, the primary difference between the 3 SIA techniques was for A/H3N2 virus mapping. Indeed, only the Froussard random PCR method performed on engorged lice with artificially contaminated human blood seemed to randomly amplify all segments (Fig 3A). Similarly, other viral reads were detected after Froussard random SIA in higher abundance than the two other random PCRs (Table 2). Additionally, the comparison of the amplicon profiles obtained after the 3 random PCRs resulted for each reference virus in important differences, highlighting the amplification bias (S2I–S2L Fig). Due to the necessity of conducting random amplification after viral purification, quantitative analyses of the composition of resulting viromes may not reflect the initial composition of the viral communities of a given sample, and only qualitative analyses can be conducted.

When characterizing the virome of a biological sample, especially when metagenomics is used in diagnostic virology to determine the etiology of pathology, it is important to isolate the virus. Doing so allows for a clear determination of whether the cultivated virus is able to cause disease in healthy individuals, as described in Koch’s postulate in 1876 for bacteria [56] and reviewed by Rivers in 1937 for viruses [57]. Because defining the causality of a given pathology is complex, and because the isolation of viral agents remains the gold standard for conducting studies of the pathogenicity of viruses detected by metagenomics, the need to preserve the infectivity of viral particles during the metagenome analysis process has emerged. By using sucrose instead of CsCl for the gradient preparation, most of the viruses would be purified without compromising the integrity of infectious particles. This can occur with the use of CsCl gradient due to the high osmolarity of CsCl, potentially resulting in the degradation of the structure of several enveloped viruses [32–35] and loss of infectivity, as shown in S1 Fig.

## Conclusion

The resulting protocol for host-associated infectious RNA virome preparation is therefore composed of (1) a nuclease digestion of homogenized samples with Turbo DNAse, RNAse A, Benzonase, and Exonuclease I (2) a purification of viral particles on a discontinuous 66%-30% sucrose gradient ultracentrifugation at 130 000 g for 2 hours (3) a Trizol LS® RNA extraction followed by a Turbo DNAse digestion and (4) Froussard-based random RT and PCR.

The protocol reported here could easily be applied to generate RNA viral metagenomes from complex biological samples of different origins, with no loss of viral infectivity and an important elimination of contaminating host DNA and RNA after the process. Moreover, the pipeline described here allows for further virological characterizations of the described viral communities because it preserves the infectivity of viral particles and allows for the isolation of viruses.

## Supporting Information

S1 Fig Infectivity test after the virome process. A. Cytotoxicity of sucrose and CsCl on Vero cells infected with non-purified, sucrose-purified and CsCl purified CPX (day 2 post-infection, 10X objective). B. Cytopathic effects on Vero cells infected with non-purified, sucrose-purified and CsCl purified CPX (day 13 post-infection, 10X objective, dilution 1/100 of the inoculum).
(TIF)

S2 Fig Nucleic acid profiles analyzed on a 2100 Expert Agilent Analyzer. A: amplification profile using Froussard method with different amount of random primers (red: 0.05 μg/μL / blue: 0.10 μg/μL / green: 0.15 μg/μL). B: amplification profile using Wang method with different amount of random primers (red: 20 pmol / blue: 40 pmol / green: 60 pmol). C: amplification profile using Victoria method with different amount of random primers (red: 50 pmol / blue: 100 pmol / green: 150 pmol). D: amplification profile using Froussard method with different amount of random primers (red: 0.10 μg/μL / blue: 0.01 μg/μL / green: 0.001 μg/μL). E: amplification profile using Wang method with different amount of random primers (red: 40 pmol / blue: 4 pmol / green: 0.4 pmol). F: amplification profile using Victoria method with different amount of random primers (red: 100 pmol / blue: 10 pmol / green: 1 pmol). G: amplification profile with 20 (red) or 40 (blue) cycles of random PCR. H: amplification profile using Froussard method with different elongation durations (red: 1 min / blue: 3 min). I to L: amplification profile of RNA viruses according to Froussard (red), Wang (blue) and Victoria (green) random PCR.
(TIF)

S3 Fig Fluorescence microscopy of VLP after viral purification and enrichment of body lice. All images were acquired with a Leica SP5 inverted confocal microscope with 4 lasers, a 100X objective and a numerical aperture of 1.4. Scale bar means 30 μm.
(TIF)

S4 Fig Comparison of the 3 random PCR reactions in spiked lice metagenomes according to the reference genome coverage. (TIF)

S1 Table Primers used in this study. (DOCX)

## References

[1] Suttle CA. Viruses in the sea (2005) Nature 437: 356–361. 16163346 10.1038/nature04160

[2] Chiu CY. Viral pathogen discovery (2013) Curr Opin Microbiol 16: 468–478. 10.1016/j.mib.2013.05.001 23725672 PMC5964995

[3] Capobianchi MR, Giombini E, Rozera G (2013) Next-generation sequencing technology in clinical virology (2013) Clin Microbiol Infect 19: 15–22. 10.1111/1469-0691.12056 23279287

[4] Barzon L, Lavezzo E, Militello V, Toppo S, Palù G. Applications of next-generation sequencing technologies to diagnostic virology (2011) Int J Mol Sci 12: 7861–7884. 10.3390/ijms12117861 22174638 PMC3233444

[5] Belák S, Karlsson OE, Blomström AL, Berg M, Granberg F. New viruses in veterinary medicine, detected by metagenomic approaches (2013) Vet Microbiol 165: 95–101. 10.1016/j.vetmic.2013.01.022 23428379

[6] Blomström AL. Viral metagenomics as an emerging and powerful tool in veterinary medicine (2011) Vet Q. 10.1080/01652176.2011.604971 22029881

[7] Temmam S, Davoust B, Berenger JM, Raoult D, Desnues C. Viral metagenomics on animals as a tool for the detection of zoonoses prior to human infection? (2014) Int J Mol Sci 15: 10377–10397. 10.3390/ijms150610377 24918293 PMC4100157

[8] Roossinck MJ. Plant virus metagenomics: biodiversity and ecology (2012) Annu Rev Genet 46: 359–369. 10.1146/annurev-genet-110711-155600 22934641

[9] Ng TF, Duffy S, Polston JE, Bixby E, Vallad GE, Breitbart M. Exploring the diversity of plant DNA viruses and their satellites using vector-enabled metagenomics on whiteflies (2011) PLos One. 10.1371/journal.pone.0019050 21544196 PMC3081322

[10] Cox-Foster DL, Conlan S, Holmes EC, Palacios G, Evans JD, Moran NA, et al A metagenomic survey of microbes in honey bee colony collapse disorder (2007) Science. 318: 283–287. 17823314 10.1126/science.1146498

[11] Granberg F, Vicente-Rubiano M, Rubio-Guerri C, Karlsson OE, Kukielka D, Belák S, et al Metagenomic detection of viral pathogens in Spanish honeybees: co-infection by Aphid Lethal Paralysis, Israel Acute Paralysis and Lake Sinai Viruses (2013) PLos One. 10.1371/journal.pone.0057459 23460860 PMC3583878

[12] Popgeorgiev N, Temmam S, Raoult D, Desnues C. Describing the silent human virome with an emphasis on giant viruses (2013) Intervirology 56: 395–412. 10.1159/000354561 24157886

[13] Rosario K, Breitbart M. Exploring the viral world through metagenomics (2011) Curr Opin Virol 1: 289–297. 10.1016/j.coviro.2011.06.004 22440785

[14] Fancello L, Trape S, Robert C, Boyer M, Popgeorgiev N, Raoult D, et al Viruses in the desert: a metagenomic survey of viral communities in four perennial ponds of the Mauritanian Sahara (2013) ISME J 7: 359–369. 10.1038/ismej.2012.101 23038177 PMC3554411

[15] Whon TW, Kim MS, Roh SW, Shin NR, Lee HW, Bae JW. Metagenomic characterization of airborne viral DNA diversity in the near-surface atmosphere (2012) J Virol 86: 8221–8231. 10.1128/JVI.00293-12 22623790 PMC3421691

[16] Djikeng A, Kuzmickas R, Anderson NG, Spiro DJ. Metagenomic analysis of RNA viruses in a fresh water lake (2009). PLos One. 10.1371/journal.pone.0007264 19787045 PMC2746286

[17] Angly FE, Felts B, Breitbart M, Salamon P, Edwards RA, Carlson C, et al The marine viromes of four oceanic regions (2006) PLos Biol. 4: e368 17090214 10.1371/journal.pbio.0040368 PMC1634881

[18] Thurber RV (2011) Methods in Viral Metagenomics In: de Bruijn FJ, editor. Handbook of Molecular Microbial Ecology, Volume II: Metagenomics in Different Habitats. Wiley-Blackwell pp 15–24.

[19] Thurber RV, Haynes M, Breitbart M, Wegley L, Rohwer F. Laboratory procedures to generate viral metagenomes (2009) Nat Protoc 4: 470–483. 10.1038/nprot.2009.10 19300441

[20] Hall RJ, Wang J, Todd AK, Bissielo AB, Yen S, Strydom H, et al Evaluation of rapid and simple techniques for the enrichment of viruses prior to metagenomic virus discovery (2014) J Virol Methods 195: 194–204. 10.1016/j.jviromet.2013.08.035 24036074 PMC7113663

[21] Stang A, Korn K, Wildner O, Uberla K. Characterization of virus isolates by particle-associated nucleic acid PCR (2005) J Clin Microbiol 43: 716–720. 15695669 10.1128/JCM.43.2.716-720.2005 PMC548055

[22] Weynberg KD, Wood-Charlson EM, Suttle CA, van Oppen MJ. Generating viral metagenomes from the coral holobiont (2014) Front Microbiol. 10.3389/fmicb.2014.00206 24847321 PMC4019844

[23] Drosten C, Göttig S, Schilling S, Asper M, Panning M, Schmitz H, et al Rapid detection and quantification of RNA of Ebola and Marburg viruses, Lassa virus, Crimean-Congo hemorrhagic fever virus, Rift Valley fever virus, dengue virus, and yellow fever virus by real-time reverse transcription-PCR (2002) J Clin Microbiol 40: 2323–2330. 12089242 10.1128/JCM.40.7.2323-2330.2002 PMC120575

[24] Watkins-Riedel T, Woegerbauer M, Hollemann D, Hufnagl P. Rapid diagnosis of enterovirus infections by real-time PCR on the LightCycler using the TaqMan format (2002) Diagn Microbiol Infect Dis 42: 99–105. 11858904 10.1016/s0732-8893(01)00330-3

[25] van Elden LJ, Nijhuis M, Schipper P, Schuurman R, van Loon AM. Simultaneous detection of influenza viruses A and B using real-time quantitative PCR (2001) J Clin Microbiol 39: 196–200. 11136770 10.1128/JCM.39.1.196-200.2001 PMC87701

[26] Ninove L, Nougairede A, Gazin C, Thirion L, Delogu I, Zandotti C, et al RNA and DNA bacteriophages as molecular diagnosis controls in clinical virology: a comprehensive study of more than 45,000 routine PCR tests (2011) PLos One. 10.1371/journal.pone.0016142 21347398 PMC3036576

[27] Breitbart M, Rohwer F. Method for discovering novel DNA viruses in blood using viral particle selection and shotgun sequencing (2005) Biotechniques 39: 729–736. 16312220 10.2144/000112019

[28] Sangaré AK, Boutellis A, Drali R, Audoly G, Weber P, Rolain JM, et al Doxycycline kills human lice through its activity on their bacterial symbiont (2015) Int J Antimicrob Agents 45: 675–676. 10.1016/j.ijantimicag.2015.02.008 25836018

[29] Froussard P. A random-PCR method (rPCR) to construct whole cDNA library from low amounts of RNA (1992) Nucleic Acids Res 20: 2900 1614887 10.1093/nar/20.11.2900 PMC336952

[30] Wang D, Coscoy L, Zylberberg M, Avila PC, Boushey HA, Ganem D, et al Microarray-based detection and genotyping of viral pathogens (2002) Proc Natl Acad Sci U S A 99: 15687–15692. 12429852 10.1073/pnas.242579699 PMC137777

[31] Victoria JG, Kapoor A, Dupuis K, Schnurr DP, Delwart EL. Rapid identification of known and new RNA viruses from animal tissues (2008) PLoS Pathog. 10.1371/journal.ppat.1000163 18818738 PMC2533695

[32] Trudel M, Payment P (1989) Purification et analyse de virus par ultracentrifugation In: Manuel de Techniques Virologiques, Presses de l’Université du Québec.

[33] King AMQ, Adams MJ, Carstens EB, Lefkowitz EJ (2011) Virus Taxonomy: ninth report of the International Committee on Taxonomy of Viruses Elsevier Academic Press.

[34] Lawrence JE, Steward GF (2010) Purification of viruses by centrifugation In: Wilhelm SW, Weinbauer MG and Suttle CA, editors. Manual of Aquatic Viral Ecology. ASLO Pp 166–181.

[35] Tidona CA, Darai G (2001). The Springer Index of Viruses. Springer.

[36] Kleiner M, Hooper LV, Duerkop BA. Evaluation of methods to purify virus-like particles for metagenomic sequencing of intestinal viromes (2015) BMC Genomics. 10.1186/s12864-014-1207-4 25608871 PMC4308010

[37] Cheval J, Sauvage V, Frageul L, Dacheux L, Guignon G, Dumey N, et al Evaluation of high-throughput sequencing for identifying known and unknown viruses in biological samples (2011) J Clin Microbiol. 49: 3268–3275. 10.1128/JCM.00850-11 21715589 PMC3165575

[38] Frey KG, Herrera-Galeano JE, Redden CL, Luu TV, Servetas SL, Mateczun AJ, et al Comparison of three next-generation sequencing platforms for metagenomic sequencing and identification of pathogens in blood (2014) BMC Genomics. 10.1186/1471-2164-15-96 24495417 PMC3922542

[39] Phan TG, Vo NP, Bonkoungou IJ, Kapoor A, Barro N, O'Ryan M, et al Acute diarrhea in West African children: diverse enteric viruses and a novel parvovirus genus (2012) J Virol 86: 11024–11030. 22855485 10.1128/JVI.01427-12 PMC3457132

[40] Ng TF, Marine R, Wang C, Simmonds P, Kapusinszky B, Bodhidatta L, et al High variety of known and new RNA and DNA viruses of diverse origins in untreated sewage (2012) J Virol 86: 12161–12175. 10.1128/JVI.00869-12 22933275 PMC3486453

[41] Colson P, de Lamballerie X, Fournous G, Raoult D. Reclassification of giant viruses composing a fourth domain of life in the new order Megavirales (2012) 55: 321–332. 10.1159/000336562 22508375

[42] Daly GM, Bexfield N, Heaney J, Stubbs S, Mayer AP, Palser A, et al A viral discovery methodology for clinical biopsy samples utilising massively parallel next generation sequencing (2011) PLos One. 10.1371/journal.pone.0028879 22216131 PMC3244418

[43] Yang Y, Griffiths MW. Enzyme treatment reverse transcription-PCR to differentiate infectious and inactivated F-specific RNA phages (2014) Appl Environ Microbiol. 80: 3334–3340. 10.1128/AEM.03964-13 24657854 PMC4018871

[44] Hurwitz BL, Deng L, Poulos BT, Sullivan MB. Evaluation of methods to concentrate and purify ocean virus communities through comparative, replicated metagenomics (2013) Environ Microbiol 15: 1428–1440. 10.1111/j.1462-2920.2012.02836.x 22845467 PMC3655615

[45] Sachsenröder J, Twardziok S, Hammerl JA, Janczyk P, Wrede P, Hertwig S, et al Simultaneous identification of DNA and RNA viruses present in pig faeces using process-controlled deep sequencing (2012) PLos One. 10.1371/journal.pone.0034631 22514648 PMC3326065

[46] Marston DA, McElhinney LM, Ellis RJ, Horton DL, Wise EL, Leech SL, et al Next generation sequencing of viral RNA genomes (2013) BMC Genomics. 10.1186/1471-2164-14-444 23822119 PMC3708773

[47] Maillard P, Walic M, Meuleman P, Roohvand F, Huby T, Le Goff W, et al Lipoprotein lipase inhibits hepatitis C virus (HCV) infection by blocking virus cell entry (2011) PLoS One. 10.1371/journal.pone.0026637 22039521 PMC3198807

[48] Kohl C, Brinkmann A, Dabrowski PW, Radonić A, Nitsche A, Kurth A. Protocol for metagenomic virus detection in clinical specimens (2015) Emerg Infect Dis. 21: 48–57. 10.3201/eid2101.140766 25532973 PMC4285256

[49] Mathieson W, Thomas GA. Simultaneously extracting DNA, RNA, and protein using kits: is sample quantity or quality prejudiced? (2013) Anal Biochem 433: 10–18. 10.1016/j.ab.2012.10.006 23068038

[50] Kim KH, Bae JW. Amplification methods bias metagenomic libraries of uncultured single-stranded and double-stranded DNA viruses (2011) Appl Environ Microbiol 77: 7663–7668. 10.1128/AEM.00289-11 21926223 PMC3209148

[51] Marine R, McCarren C, Vorrasane V, Nasko D, Crowgey E, Polson SW, et al Caught in the middle with multiple displacement amplification: the myth of pooling for avoiding multiple displacement amplification bias in a metagenome (2014) Microbiome. 10.1186/2049-2618-2-3 24475755 PMC3937105

[52] Berthet N, Reinhardt AK, Leclercq I, van Ooyen S, Batéjat C, Dickinson P, et al Phi29 polymerase based random amplification of viral RNA as an alternative to random RT-PCR (2008) BMC Mol Biol. 10.1186/1471-2199-9-77 18771595 PMC2535778

[53] Delwart EL. Viral metagenomics (2007) Rev Med Virol 17:115–131. 17295196 10.1002/rmv.532 PMC7169062

[54] Karlsson OE, Belák S, Granberg F. The effect of preprocessing by sequence-independent, single-primer amplification (SISPA) on metagenomic detection of viruses (2013) Biosecur Bioterror 11 Suppl 1: 227–234. 10.1089/bsp.2013.0008 23971810

[55] Rosseel T, Van Borm S, Vandenbussche F, Hoffmann B, van den Berg T, Beer M, et al The origin of biased sequence depth in sequence-independent nucleic acid amplification and optimization for efficient massive parallel sequencing (2013) PLos One. 10.1371/journal.pone.0076144 24086702 PMC3784409

[56] Koch R. Investigations into bacteria: V. The etiology of anthrax, based on the ontogenesis of *Bacillus anthracis* (1876) Cohns Beitrage zur Biologie der Pflanzen 2: 277–310.

[57] Rivers TM. Viruses and Koch’s Postulates. (1937) J Bacteriol 33: 1–12. 16559982 10.1128/jb.33.1.1-12.1937 PMC545348

