# Supplementary figures and images for: Host-Associated Metagenomics: A Guide to Generating Infectious RNA Viromes
Source: PLoS One. 2015 Oct 2;10(10):e0139810. doi: 10.1371/journal.pone.0139810 (PMC4592258; doi:10.1371/journal.pone.0139810)

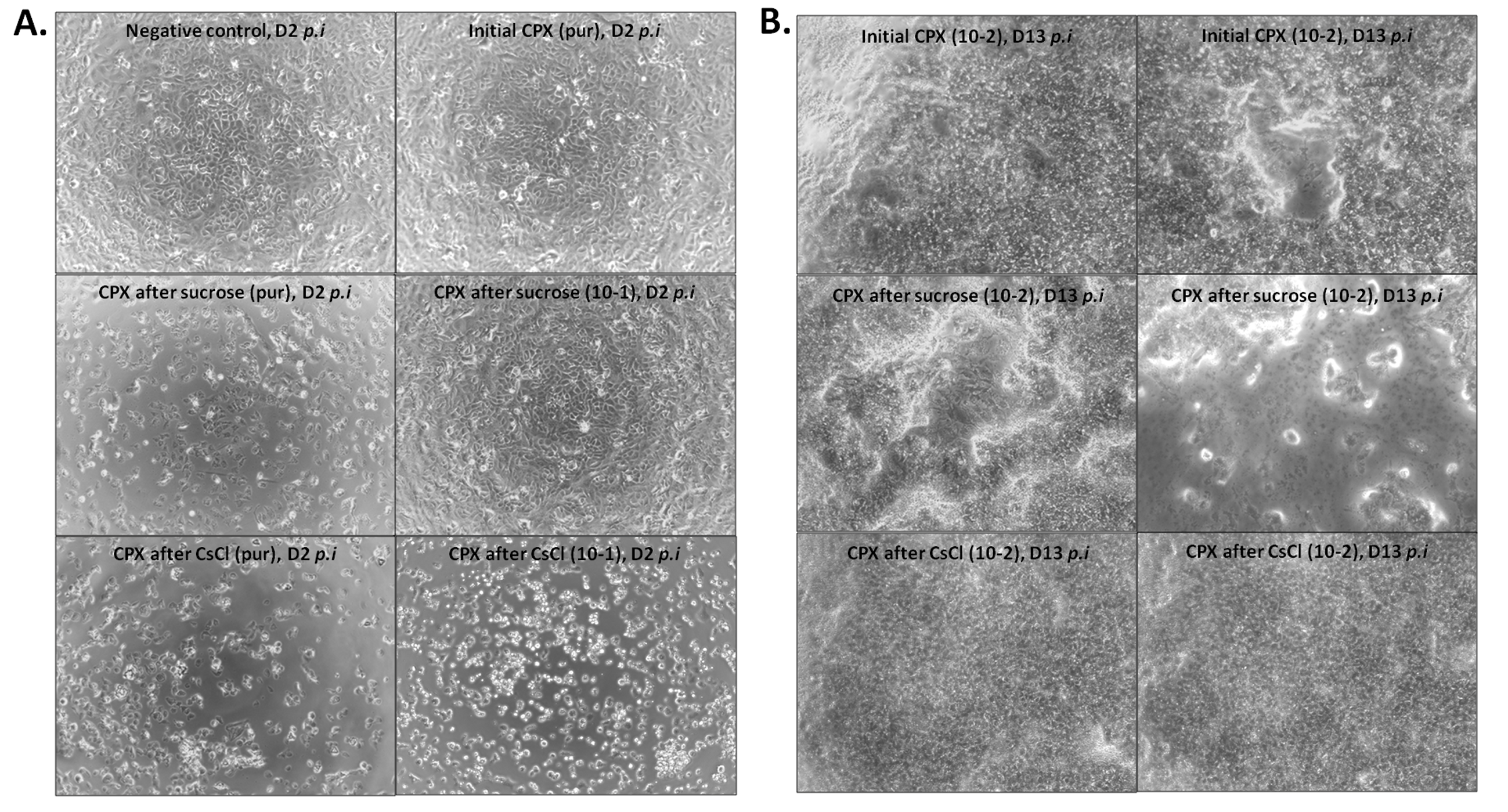

Supplement: S1 Fig — A. Cytotoxicity of sucrose and CsCl on Vero cells infected with non-purified, sucrose-purified and CsCl purified CPX (day 2 post-infection, 10X objective). B. Cytopathic effects on Vero cells infected with non-purified, sucrose-purified and CsCl purified CPX (day 13 post-infection, 10X objective, dilution 1/100 of the inoculum). (TIF) [file pone.0139810.s001.tif]

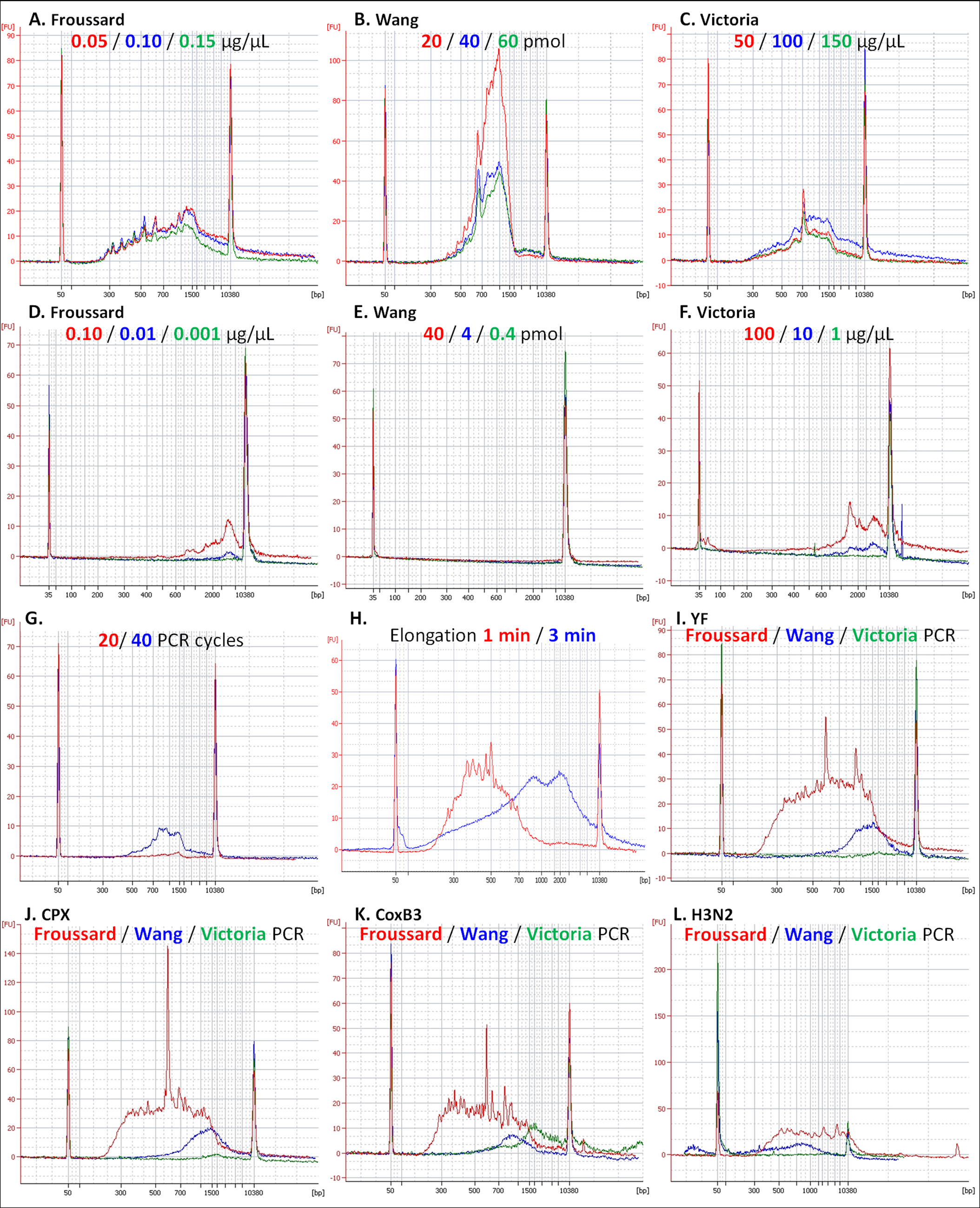

Supplement: S2 Fig — A: amplification profile using Froussard method with different amount of random primers (red: 0.05 μg/μL / blue: 0.10 μg/μL / green: 0.15 μg/μL). B: amplification profile using Wang method with different amount of random primers (red: 20 pmol / blue: 40 pmol / green: 60 pmol). C: amplification profile using Victoria method with different amount of random primers (red: 50 pmol / blue: 100 pmol / green: 150 pmol). D: amplification profile using Froussard method with different amount of random primers (red: 0.10 μg/μL / blue: 0.01 μg/μL / green: 0.001 μg/μL). E: amplification profile using Wang method with different amount of random primers (red: 40 pmol / blue: 4 pmol / green: 0.4 pmol). F: amplification profile using Victoria method with different amount of random primers (red: 100 pmol / blue: 10 pmol / green: 1 pmol). G: amplification profile with 20 (red) or 40 (blue) cycles of random PCR. H: amplification profile using Froussard method with different elongation durations (red: 1 min / blue: 3 min). I to L: amplification profile of RNA viruses according to Froussard (red), Wang (blue) and Victoria (green) random PCR. (TIF) [file pone.0139810.s002.tif]

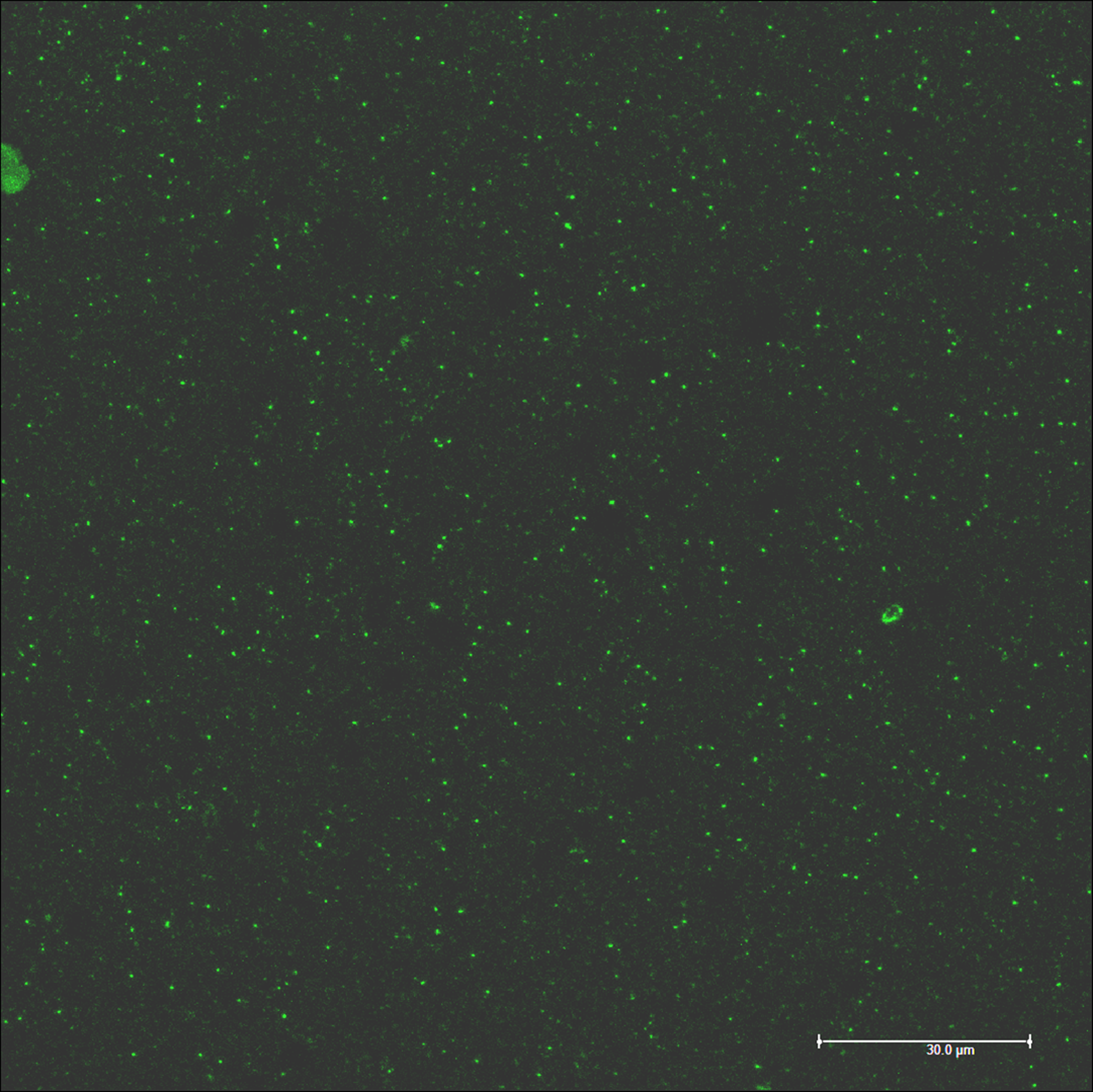

Supplement: S3 Fig — All images were acquired with a Leica SP5 inverted confocal microscope with 4 lasers, a 100X objective and a numerical aperture of 1.4. Scale bar means 30 μm. (TIF) [file pone.0139810.s003.tif]

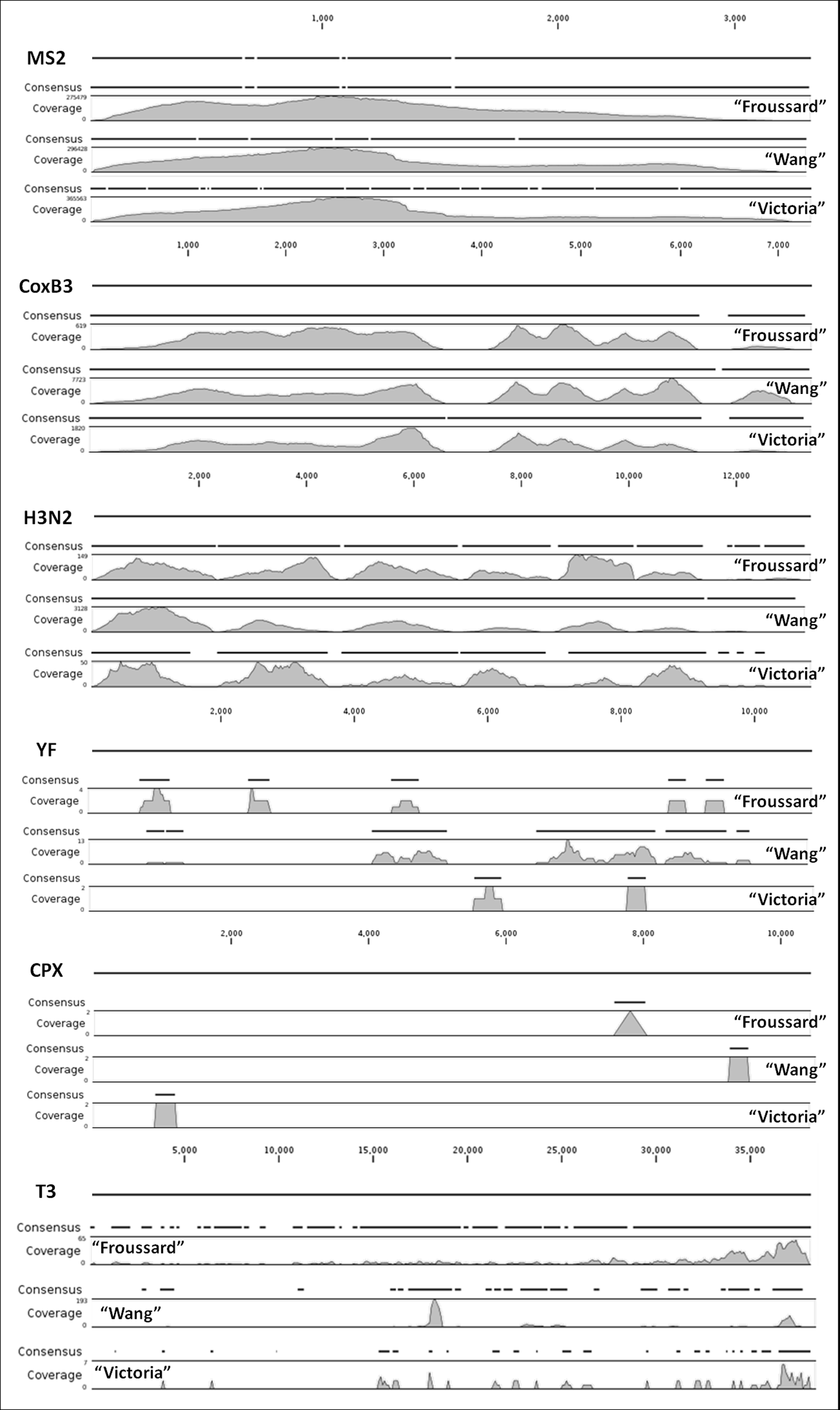

Supplement: S4 Fig — (TIF) [file pone.0139810.s004.tif]
