# Supplementary material for: Host-Associated Metagenomics: A Guide to Generating Infectious RNA Viromes
Source: PLoS One. 2015 Oct 2;10(10):e0139810. doi: 10.1371/journal.pone.0139810 (PMC4592258; doi:10.1371/journal.pone.0139810)
Supplement: S1 Table — (DOCX) [file pone.0139810.s005.docx]

| **Virus** | **Primer** | **Sequence (5’ 🡪 3’)** | **Reference** |
| --- | --- | --- | --- |
| YF | forward | AATCGAGTTGCTAGGCAATAAACAC | 23 |
|  | reverse | TCCCTGAGCTTTACGACCAGA |  |
| CoxB3 | forward | CCCCTGAATGCGGCTAATCC | 24 |
|  | reverse | ATTGTCACCATAAGCAGCCA |  |
| H3N2 | forward | CATYCTGTTGTATATGAGGCCCAT | 25 |
|  | reverse | GGACTGCAGCGTAGACGCTT |  |
| MS2 | forward | GTTCCCTACAACGAGCCTAAATTC | 26 |
|  | reverse | CTCTGAGAGCGGCTCTATTGGT |  |
| T4 | forward | CCATCCATAGAGAAAATATCAGAACGA | 26 |
|  | reverse | CGCTGGGAAAAGAGGAATTATTTA |  |
| CPX | forward | TGGCAGAGAATGGTATAGTAGG | This study |
|  | reverse | GCTTTCCCATATCAGACTTCC |  |
| 18S | forward | CCGCAGCTAGGAATAATGGAATAGGAC | 27 |
|  | reverse | ITTAGCATGCCAGAGTCTCGTTCGT |  |
| YF (virome-based) | forward | TGGTGAAGTTTCATGGGAAGAGG | This study |
|  | reverse | CCAAGATGGAATCAACTTCTTGCC |  |
| H3N2 (virome-based) | forward | TGGATCAAGTGAGAGAAAGTCGG | This study |
|  | reverse | CTCATTTGAGGCAATTTGTACTCC |  |
